# Supplementary material for: Adherence to Medication in Neurogeriatric Patients: Insights from the NeuroGerAd Study
Source: J Clin Med. 2022 Sep 13;11(18):5353. doi: 10.3390/jcm11185353 (PMC9501565; doi:10.3390/jcm11185353)
Supplement: Supplementary file 1 [file jcm-11-05353-s001.zip › jcm-1874398-supplementary.pdf]

Figure S1. Screening procedure

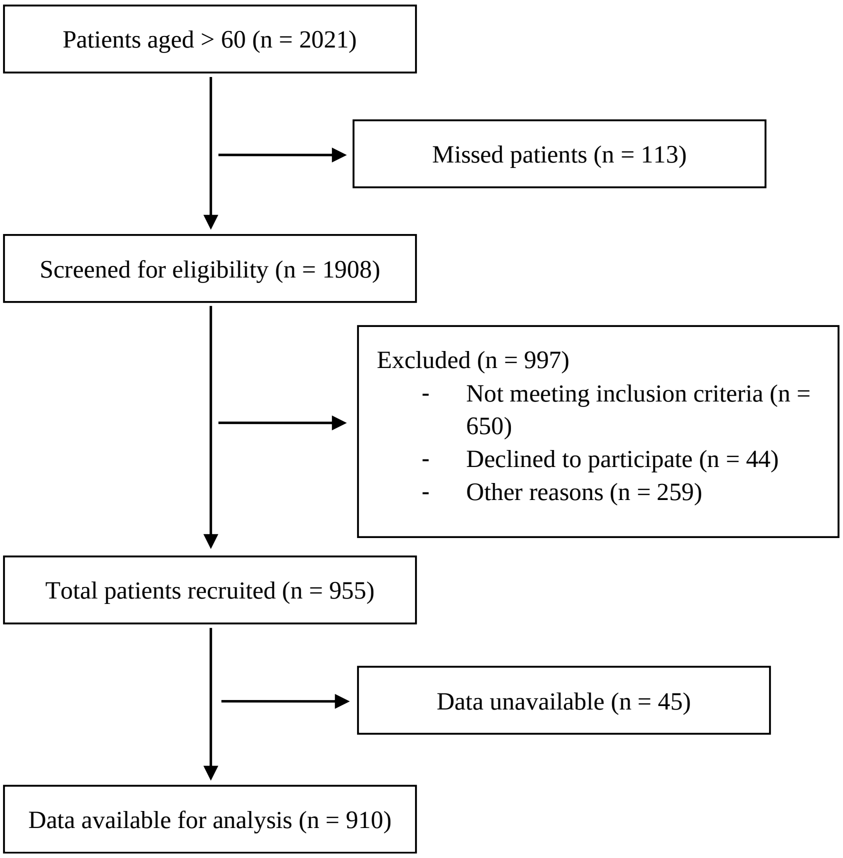

Table S1: Assessments and questionnaires

Domain	Score	Rating	Reference
Depression	Beck-Depression-Inventory II (BDI II)	The BDI-II is scored by summing the highest ratings for each of the 21 symptoms. Each symptom is rated for the past two weeks including the present day on a four-point rating scale (0–3). Sum scores range from 0 to 63. The following severity levels are suggested in the manual: Scores between 0 and 13 indicate minimal, between 14 and 19 mild, between 20 and 28 moderate, and between 29 and 63 severe depression	(1, 2)
Cognition	Montreal Cognitive Assessment (MoCA)	The MoCA is a common screen that targets the differentiation between normal aging and MCI and has gained worldwide traction among healthcare professionals. The MoCA test is straightforward to administer and easy to access (downloaded without cost from www.mocatest.org). It is scored out of 30 points, with higher scores reflecting better performance. The MoCA examines the following cognitive abilities: visuospatial/executive function, naming, episodic memory, attention, language, abstraction, and orientation. Nasreddine *et al.* (2005) suggested a cutoff score of 26, with those scoring 25 or below suspected of having MCI. A current meta-analysis indicated that a cutoff score of 23 on the MoCA offered better diagnostic accuracy than the originally recommended cutoff score of 26 (3) .	(3, 4)
Mobility	Timed up and Go Test (TUG-test)	The timed up and go test (TUG-test) is an effective method of assessing mobility and quantifying locomotor performance. The TUG-test is objective, quick and easy to perform. The test includes basic mobility skills, such as rising from a chair, walking 3 meters, turning and sitting down on the same chair. Subjects were observed and timed from the instant they rose from an armchair,walked 3 metres, and returned to a fully seated position in the chair. Subjects wore their regular footwear and were allowed to use the arms of the chair to get up. Subjects began the test on the word, 'go' and were instructed to 'walk at a comfortable fast and secure pace'. The score is the time in seconds that the subject needed to complete the test.	(5)
Personality	Big Five Inventory 10 (BFI-10)	The BFI-10 has five subscales with two bidirectional items for each of the big-five personality factors. The items are rated on a five-point Likert scale wherein the subjects choose from responses ranging from "strongly disagree "to "strongly agree". Scale scores are then calculated as the participant's mean response.	(6)<br>(7)
Autonomy support/ health care climate	Health Care Climate Questionnaire (HCCQ)	The HCCQ is made up of 15 items using a Likert scale ranging from 1 = strongly disagree to 7 = strongly agree, with item 13 being coded in reverse. The HCCQ analyses patients' perception of support for autonomy, competence, and relatedness, enabling the researcher to gauge a patients' perception of healthcare providers' support in preserving their autonomy. The score is calculated s a mean score, with higher scores indicating a higher level of autonomy support.	(8)
Health related quality of life	Short Form Health Survey (SF-36)	The SF-36 is a disease-unspecific questionnaire to assess health-related quality of life in the last 4 weeks prior to testing. It encompasses 8 different domains in 36 items, including problems regarding both physical and social activity due to health, limitations in daily life due to physical or emotional problems, pain, mental health, vitality, and general health perception. Each domain is analyzed as the weighted sum of the corresponding items, with lower scores indicating less disability.	(9)

Adherence to medication	Stendal Adherence to Medication Score (SAMS)	The questionnaire comprises 18 items adding up to a cumulative adherence scale, with 0 indicating complete adherence and 72 complete non-adherence. Different aspects of adherence are covered, such as intentional modification of medication, lack of knowledge and forgetting to take the medication.	(10)

Table S2. Specification of neurological diagnoses

**Diagnosis**	**n**	**%**
PD	215	23.6
Atypical/Secondary PD	45	4.9
Tremor, Dystonia, Other	43	4.7
Acute infarction	173	19.0
Chronic neurovascular problem	25	2.7
Other neurovascular diagnosis	34	3.7
Epilepsy, idiopathic	4	0.4
Structural epilepsy	19	2.1
Other epileptic problem/unclassified	25	2.7
ALS	21	2.3
Other neuromuscular disease	25	2.7
Peripheral neuropathy	123	13.5
OSAS	30	3.3
Spinal problems	18	2.0
Others	110	12.1
Total	910	100.0

Table S3. Principal Component Analysis of Stendal Adherence to Medication Score (SAMS)

Item	Factor with factor loadings		
	**Modification**	**Missing knowledge**	**Forgetting**
If you think you have side effects due to of the medications (such as tremors, nausea etc.), do you not take the medication for a while, i.e. take a break?	0.867		
If you think you have side effects due to of the medications (such as tremors, nausea etc.), do you reduce the dose without consulting a doctor?	0.795		
If you feel you have to take too many tablets, do you stop taking those medications you consider to be less important than the others without consulting your doctor?	0.791		
Do you stop taking your medication if you sometimes feel worse after taking the medication?	0.677		
Do you stop taking your medication when you feel better?	0.673		
Do you deliberately not take medications you do not consider important, but take the rest?	0.584		
Do you take any wrong or other/unprescribed medications (such as those of your partner)?	0.558		
Do you know the dosages of your medication?		0.857	
Do you know the names of medications you are taking?		0.790	
Do you know the reason for taking your medication?		0.761	
Are you familiar with the timing for taking the medication?		0.727	
If you forget or omit your medication, do you forget it in the evening?			0.744
Do you forget to take your medication?			0.738
If you forget or omit your medication, do you forget it at noon?			0.708
If you forget or omit your medication, do you forget it in the morning?			0.669
**Eigenvalue**	**3.756**	**2.57**	**2.343**
**Variance explained**	**25.038**	**17.131**	**15.62**
**Cronbachs Alpha**	**0.851**	**0.798**	**0.731**

Both the Bartlett test ($p < 0.001$) and the Kaiser–Meyer–Olkin Measure of Sampling Adequacy ($p = 0.85$) indicated that the variables were suitable for factor analysis. Three items, items 4 (Do you take your medication regularly?), 7 (Are you untroubled about taking the medication?), and 18 (If you take medication from a syringe or in a weekly tablet, have you ever forgotten it?), exhibited a low communality score and were removed from the analysis.

Supplement Table S4 A-C: Predictors of Stendal Adherence to Medication Score (SAMS) subfactors

A. Predictors of the factor Modification

	**coefficient**	**Standard error**	**95% CI lower limit**	**95% CI upper limit**	**p**
Constant	0.321	0.652	-0.960	1.602	0.623
Factor-1	0	.	.	.	.
BFI extraversion	0.151	0.199	-0.241	0.543	0.449
BFI conscientiousness	0.168	0.179	-0.185	0.521	0.350
BFI neuroticism	-0.205	0.217	-0.632	0.222	0.345
BFI openness	0.084	.206	-.320	0.488	0.683
BFI agreeableness	0	.	.	.	.
Gender female	-0.062	0.095	-0.249	0.125	0.515
Gender male	0	.	.	.	.
Diagnosis movement disorder	0.018	0.132	-0.241	0.277	0.890
Diagnosis cerebrovascular disorder	-0.098	0.146	-0.384	0.189	0.503
Diagnosis epilepsy	-0.237	0.246	-0.720	0.246	0.335
Diagnosis neuromuscular	0.041	0.142	-0.237	0.320	0.770
Diagnosis others	0	.	.	.	.
Living situation alone	0.033	0.111	-0.185	0.251	0.764
Living situation not alone	0	.	.	.	.
Education level high	-0.064	0.120	-0.300	0.172	0.593
Education level middle	-0.116	0.121	-0.355	0.123	0.340
Education level low	0	.	.	.	.
Age	-0.011	0.006	-0.023	0.001	0.070
**number of medications/day**	**-0.044**	**0.013**	**-0.070**	**-0.018**	**0.001**
**BDI**	**0.047**	**0.007**	**0.033**	**0.060**	**0.000**
HCCQ-D	-0.043	0.043	-0.126	0.041	0.315
MoCA	0.012	0.013	-0.014	0.038	0.370
TuG	0.018	0.011	-0.003	0.039	0.087

B. Predictors of the factor Missing Knowledge

	**coefficient**	**Standard error**	**95% CI lower limit**	**95% CI upper limit**	**p**
Constant	0.276	0.552	-0.809	10.362	0.617
Factor-2	0	.	.	.	.
BFI extraversion	0.074	0.169	-0.258	0.406	0.661
BFI conscientiousness	0.100	0.152	-0.199	0.399	0.510
BFI neuroticism	0.166	0.184	-0.196	0.528	0.367
BFI openness	0.119	0.174	-0.224	0.461	0.496
BFI agreeableness	0	.	.	.	.
**Gender female**	**-0.164**	**0.081**	**-0.322**	**-0.005**	**0.043**
Gender male	0	.	.	.	.
Diagnosis movement disorder	0.001	0.112	-0.218	0.221	0.992
Diagnosis cerebrovascular disorder	0.045	0.124	-0.198	0.288	0.715
Diagnosis epilepsy	0.131	0.208	-0.279	0.540	0.531
Diagnosis neuromuscular	-0.072	0.120	-0.308	0.163	0.547
Diagnosis others	0	.	.	.	.
Living situation alone	0.011	0.094	-0.173	0.196	0.904
Living situation not alone	0	.	.	.	.
Education level high	-.114	.102	-.314	.086	.264
Education level middle	-.193	.103	-.395	.009	.061
Education level low	0	.	.	.	.
Age	0.011	0.005	0.001	0.021	0.037
**number of medications/day**	**0.055**	**0.011**	**0.033**	**0.077**	**0.000**
**BDI**	**0.012**	**0.006**	**30.477E-5**	**0.023**	**0.049**
HCCQ-D	-0.023	0.036	-0.094	0.048	0.531
**MoCA**	**-0.060**	**0.011**	**-0.083**	**-0.038**	**0.000**
TuG	0.008	0.009	-0.010	0.025	0.382

C. Predictors of the factor Forgetting

	coefficient	Standard error	95% CI lower limit	95% CI upper limit	p
Constant	-0.079	0.631	-10.319	10.162	0.901
Factor-3	0	.	.	.	.
BFI extraversion	-0.296	0.193	-0.675	0.084	0.126
BFI conscientiousness	-0.185	0.174	-0.527	0.156	0.286
BFI neuroticism	-0.230	0.210	-0.644	0.183	0.274
BFI openness	-0.128	0.199	-0.520	0.263	0.521
BFI agreeableness	0	.	.	.	.
Gender female	-0.153	0.092	-0.334	0.028	0.097
Gender male	0	.	.	.	.
Diagnosis movement disorder	0.229	0.128	-0.022	0.480	0.073
Diagnosis cerebrovascular disorder	0.012	0.141	-0.265	0.290	0.932
Diagnosis epilepsy	0.082	0.238	-0.386	0.550	0.731
Diagnosis neuromuscular	-0.149	0.137	-0.418	0.121	0.278
Diagnosis others	0	.	.	.	.
**Living situation alone**	**-0.239**	**0.107**	**-0.450**	**-0.028**	**0.026**
Living situation not alone	0	.	.	.	.
Education level high	0.193	0.116	-0.035	0.422	0.097
Education level middle	0.156	0.118	-0.075	0.387	0.186
Education level low	0	.	.	.	.
Age	0.001	0.006	-0.010	0.012	0.862
number of medications/day	0.015	0.013	-0.010	0.040	0.237
**BDI**	**0.014**	**0.007**	**0.001**	**0.028**	**0.032**
HCCQ-D	-0.068	0.041	-0.149	0.013	0.101
MoCA	0.010	0.013	-0.015	0.036	0.422
TuG	0.002	0.010	-0.018	0.022	0.868

Note: BDI = Beck's Depression Inventory II, BFI = Big Five Inventory, HCCQ = Healthcare Climate Questionnaire, MoCA = Montreal Cognitive Assessment, TuG = Timed Up and Go, CI = Confidence Interval

References

1. Beck A, Steer R, Brown G. Beck depression inventory—second edition: manual. San Antonio: The Psychological Corporation. 1996;4:561-71.
2. Hautzinger M, Keller F, Kühner C. Beck-Depressions-Inventar: Revision: Harcourt test services; 2006.
3. Carson N, Leach L, Murphy KJ. A re-examination of Montreal Cognitive Assessment (MoCA) cutoff scores. International Journal of Geriatric Psychiatry. 2018;33(2):379-88.
4. Nasreddine ZS, Phillips NA, Bédirian V, Charbonneau S, Whitehead V, Collin I, et al. The Montreal Cognitive Assessment, MoCA: a brief screening tool for mild cognitive impairment. Journal of the American Geriatrics Society. 2005;53(4):695-9.
5. Podsiadlo D, Richardson S. The timed "Up & Go": a test of basic functional mobility for frail elderly persons. Journal of the American Geriatrics Society. 1991;39(2):142-8.
6. John OP, Donahue EM, Kentle RL. The Big Five Inventory--Versions 4a and 54. Berkeley, CA: University of California, Berkeley, Institute of Personality and Social Research; 1991.
7. Rammstedt B. The 10-item Big Five Inventory: Norm values and investigation of sociodemographic effects based on a German population representative sample. European Journal of Psychological Assessment. 2007;23(3):193-201.
8. Schmidt K, Gensichen J, Petersen JJ, Szecsenyi J, Walther M, Williams G, et al. Autonomy support in primary care—validation of the German version of the Health Care Climate Questionnaire. Journal of Clinical Epidemiology. 2012;65(2):206-11.
9. Ware JE, Jr., Sherbourne CD. The MOS 36-item short-form health survey (SF-36). I. Conceptual framework and item selection. Med Care. 1992;30(6):473-83.
10. Prell T. Adherence to medication in neurogeriatric patients: an observational cross-sectional study. BMC Public Health. 2019;19(1):1012.
